# Supplementary material for: Hygiene of housing conditions and proinflammatory signals alter gene expressions in porcine adipose tissues and blood cells
Source: PeerJ. 2022 Dec 13;10:e14405. doi: 10.7717/peerj.14405 (PMC9756862; doi:10.7717/peerj.14405)
Supplement: Table S1 — Values are means ± SEM (n = 7 − 10 pigs/experimental group). 1Probability values for the effect of hygiene conditions (Hyg), genetic lines (Line), and the Hyg × Line (H × L) interaction. [file peerj-10-14405-s001.docx]

Supplementary Table 1: Relative expression of genes in perirenal adipose tissue of low (LRFI) and high (HRFI) residual feed intake pigs housed in good or poor hygiene conditions for six weeks.

|  | Good | | Poor | | *P-values^1^* | | | |
| --- | --- | --- | --- | --- | --- | --- | --- | --- |
| Genes | **LRFI** | **HRFI** | **LRFI** | **HRFI** | **Hyg** | **Line** | **H x L** |  |
| Adipocyte differentiation | | | | | | | |  |
| *CEBPA* | 0.66 ± 0.04 | 0.65 ± 0.08 | 0.57 ± 0.03 | 0.59 ± 0.05 | 0.130 | 0.980 | 0.779 |  |
| *DLK1* | 0.15 ± 0.09 | 0.34 ± 0.17 | 0.23 ± 0.06 | 0.34 ± 0.08 | 0.756 | 0.235 | 0.762 |  |
| Lipid metabolism | | | | | | | |  |
| *HSL* | 0.66 ± 0.07 | 0.72 ± 0.09 | 0.63 ± 0.04 | 0.61 ± 0.07 | 0.370 | 0.761 | 0.548 |  |
| *ACOX 1* | 0.73 ± 0.05 | 0.73 ± 0.05 | 0.68 ± 0.04 | 0.69 ± 0.07 | 0.366 | 0.920 | 0.909 |  |
| Lipid transport | | | | | | | |  |
| *FABP4* | 0.75 ± 0.05 | 0.69 ± 0.02 | 0.75 ± 0.04 | 0.67 ± 0.05 | 0.888 | 0.124 | 0.794 |  |
| Mitochondrial metabolism | | | | | | | |  |
| *COX3* | 0.75 ± 0.05 | 0.71 ± 0.07 | 0.69 ± 0.06 | 0.67 ± 0.08 | 0.396 | 0.630 | 0.922 |  |
| *UCP3* | 0.59 ± 0.07 | 0.60 ± 0.08 | 0.59 ± 0.06 | 0.60 ± 0.06 | 0.965 | 0.855 | 0.952 |  |
| *CS* | 0.71 ± 0.04 | 0.66 ± 0.04 | 0.65 ± 0.05 | 0.55 ± 0.05 | 0.100 | 0.127 | 0.590 |  |
| Oxidative stress | | | | | | | |  |
| *SOD2* | 0.71 ± 0.04 | 0.63 ± 0.04 | 0.73 ± 0.06 | 0.65 ± 0.07 | 0.732 | 0.133 | 0.979 |  |
| Adipokines | | | | | | | |  |
| *IGF2* | 0.69 ± 0.04 | 0.69 ± 0.04 | 0.70 ± 0.03 | 0.68 ± 0.05 | 0.985 | 0.807 | 0.808 |  |
| *IL-15* | 0.58 ± 0.06 | 0.59 ± 0.05 | 0.59 ± 0.07 | 0.70 ± 0.08 | 0.385 | 0.380 | 0.546 |  |

Values are means ± SEM (n = 7-10 pigs/experimental group). ^1^Probability values for the effect of hygiene conditions (Hyg), genetic lines (Line), and the Hyg x Line (H x L) interaction.
