# Supplementary material for: Hygiene of housing conditions and proinflammatory signals alter gene expressions in porcine adipose tissues and blood cells
Source: PeerJ. 2022 Dec 13;10:e14405. doi: 10.7717/peerj.14405 (PMC9756862; doi:10.7717/peerj.14405)
Supplement: Table S2 — 1Probability values for the effect of hygiene conditions (Hyg), genetic lines (Line), and their interaction. Values are means ± SEM (n = 5 − 10 pigs/experimental group). [file peerj-10-14405-s002.docx]

Supplementary Table 2: Relative expression of genes in subcutaneous adipose tissue of low (LRFI) and high (HRFI) residual feed intake pigs housed in good or poor hygiene conditions for six weeks.

|  | Good | | Poor | | *P-values^1^* | | |
| --- | --- | --- | --- | --- | --- | --- | --- |
| Genes | **LRFI** | **HRFI** | **LRFI** | **HRFI** | **Hyg** | **Line** | **Hyg x Line** |
| Adipocyte differentiation | | | | | | | |
| *CEBPA* | 0.61 ± 0.05 | 0.69 ± 0.11 | 0.67 ± 0.04 | 0.51 ± 0.04 | 0.347 | 0.490 | *0.063* |
| *DLK1* | 0.10 ± 0.03 | 0.15 ± 0.01 | 0.10 ± 0.03 | 0.23 ± 0.15 | 0.600 | 0.217 | 0.567 |
| Lipid metabolism | | | | | | | |
| *HSL* | 0.55 ± 0.06 | 0.64 ± 0.06 | 0.55 ± 0.04 | 0.55 ± 0.04 | 0.364 | 0.383 | 0.425 |
| *ACOX 1* | 0.69 ± 0.03 | 0.76 ± 0.05 | 0.74 ± 0.04 | 0.79 ± 0.05 | 0.364 | 0.150 | 0.866 |
| Lipid transport | | | | | | | |
| *FABP4* | 0.59 ± 0.05 | 0.55 ± 0.03 | 0.60 ± 0.03 | 0.53 ± 0.04 | 0.871 | 0.175 | 0.700 |
| Mitochondrial metabolism | | | | | | | |
| *COX3* | 0.67 ± 0.03 | 0.69 ± 0.05 | 0.71 ± 0.05 | 0.72 ± 0.04 | 0.373 | 0.775 | 0.897 |
| *UCP3* | 0.62 ± 0.07 | 0.62 ± 0.07 | 0.65 ± 0.03 | 0.62 ± 0.07 | 0.839 | 0.828 | 0.849 |
| *CS* | 0.71 ± 0.05 | 0.71 ± 0.04 | 0.72 ± 0.03 | 0.68 ± 0.06 | 0.826 | 0.623 | 0.664 |
| Oxidative stress | | | | | | | |
| *SOD2* | 0.70 ± 0.05 | 0.71 ± 0.05 | 0.73 ± 0.05 | 0.71 ± 0.06 | 0.791 | 0.975 | 0.767 |
| Adipokines | | | | | | | |
| *IGF2* | 0.71 ± 0.05 | 0.80 ± 0.05 | 0.67 ± 0.03 | 0.71 ± 0.05 | 0.167 | 0.157 | 0.560 |
| *IL-15* | 0.59 ± 0.07 | 0.56 ± 0.07 | 0.71 ± 0.04 | 0.62 ± 0.08 | 0.195 | 0.401 | 0.701 |

^1^Probability values for the effect of hygiene conditions (Hyg), genetic lines (Line), and their interaction. Values are means ± SEM (n = 5-10 pigs/experimental group).
